# Xiaoyao Pills Ameliorate Depression-like Behaviors and Oxidative Stress Induced by Olfactory Bulbectomy in Rats via the Activation of the PIK3CA-AKT1-NFE2L2/BDNF Signaling Pathway

**DOI:** 10.3389/fphar.2021.643456

**Published:** 2021-04-15

**Authors:** Yafei Ji, Jie Luo, Jiuseng Zeng, Yang Fang, Rong Liu, Fei Luan, Nan Zeng

**Affiliations:** ^1^ State Key Laboratory of Southwestern Chinese Medicine Resources, Chengdu University of Traditional Chinese Medicine, Chengdu, China; ^2^ Department of Pharmacology, School of Pharmacy, Chengdu University of Traditional Chinese Medicine, Chengdu, China

**Keywords:** xiaoyao pills, depression, olfactory bulbectomized rats, oxidative stress, inflammatory response

## Abstract

Numerous studies have revealed that oxidative stress is closely associated with the occurrence and development of depression. Xiaoyao Pills (XYW) are included in the Chinese Pharmacopoeia and are frequently used for treating anxiety and depression by smoothing the liver, strengthening the spleen, and nourishing the blood. However, the antidepressant effects of XYW have not yet been thoroughly investigated. The objective of our study was to investigate the antidepressant-like effects of XYW and the underlying molecular mechanism in the olfactory bulbectomized (OB) rat model of depression using the open field test (OFT), sucrose preference test (SPT), splash test (ST), and novelty suppressed feeding test (NSFT). Results showed that XYW (0.93 and 1.86 g·kg^−1^) significantly alleviated depression-like behaviors in rats, which was indicated by increased sucrose preference in the SPT, prolonged grooming time in the ST, decreased horizontal movement in the OFT, and shorter feeding latency in the NSFT. In addition, XYW treatment dramatically reversed the reduced activity of superoxide dismutase and the decreased level of glutathione, while also lowering levels of malondialdehyde, an inflammatory mediator (nitric oxide), and pro-inflammatory cytokines (interleukin-6 and 1β) in the serum and cortex of OB rats. Mechanistically, XYW induced marked upregulation of mRNA and protein expression levels of NFE2L2, KEAP1, GPX3, HMOX1, SOD1, NQO1, OGG1, PIK3CA, p-AKT1/AKT1, NTRK2, and BDNF, and downregulation of ROS in the cortex and hippocampus via the activation of the NFE2L2/KEAP1, PIK3CA/AKT1, and NTRK2/BDNF pathways. These findings suggest that XYW exert antidepressant-like effects in OB rats with depression-like symptoms, and these effects are mediated by the alleviation of oxidative stress and the enhancement of neuroprotective effects through the activation of the PIK3CA-AKT1-NFE2L2/BDNF signaling pathways.

## Introduction

Depression is one of the most common mental illnesses and is characterized by low mood, sleep disturbances, loss of energy, change in appetite, constipation, decreased concentration, and risk of suicide (Cui, 2015). It has become increasingly clear that oxidative stress may play a crucial role in the activation of intracellular signaling pathways that are thought to be involved in various psychiatric disorders (Michael et al., 2011). Long-term exposure to stress may lead to changes in the structure and function of the brain, which can lead to cognitive deficits and an increased risk of developing a psychiatric disorder (Lupien et al., 2009; Dragana et al., 2016). It has been suggested that environmental stressors may have a significant impact on the generation of reactive oxygen species (ROS) in the brain (Manoli et al., 2000; Fontella et al., 2005) disruptions in the balance between the clearance and production of active free radicals (i.e., ROS) in the body induce an oxidative stress reaction, which leads to macromolecular oxidation (e.g., lipids, DNA, and proteins) (Tanja et al., 2012), oxidation defense system damage (e.g. glutathione [GSH], and superoxide dismutase [SOD]) (Herbet et al., 2017), and inflammation (Hovatta et al., 2010). Increased levels of ROS in the blood have been reported in patients with major depression (Bilici et al., 2001). Moreover, previous studies have confirmed that there is a close relationship between excessive elevation of ROS levels and the pathogenesis of depression (Du et al., 2016; Herbet et al., 2017).

Clinically, traditional Chinese medicines (TCMs) are commonly used as complementary and alternative therapies for the treatment of depression. Recently, numerous studies have demonstrated that TCMs are effective in relieving symptoms of depression in both patients and animal models via various complex mechanisms (Wang Y. et al., 2017). Xiaoyao Pills (XYW) are a modified form of the commonly prescribed Xiaoyao powder, which contains eight herbs in various ratios (*Bupleurum chinense DC*., *Angelica sinensis Diels.*, *Paeonia lactiflora Pall.*, *Atractylodes macrocephala Koidz.*, *Mentha haplocalyx Briq.*, *Poria cocos Wolf.*, *Glycyrrhiza uralensis Fisch.*, *Zingiber officinale Rosc.* in ratios of 9:9:9:9:1.5:9:4.5:9, respectively). Because it is included in the China Pharmacopoeia Commision, 2020 Edition, XYW has the advantages of an established preparation technology and strict quality control compared with Xiaoyao powder. According to the TCM theory, the pathogenesis of depression is linked to liver-*qi* stagnation, blood stasis, and a deficiency of the spleen-*qi* (Zhang et al., 2005). Xiaoyao powder is thought to treat and prevent depressive syndromes by effectively smoothing the liver, nourishing blood, and strengthening the spleen. In our previous studies, we demonstrated that Xiaoyao powder exerts definitive anti-depressive effects by regulating the level and function of serotonin (Xiong et al., 2007a; Xiong et al., 2007b), improving neuroinflammation (Shi et al., 2019a; Fang et al., 2020), promoting synaptic plasticity (Shi et al., 2018; Shi et al., 2019a; Shi et al., 2019b; Shi B. et al., 2019), reversing decreases in neurotrophic factor (Wang et al., 2018c), and reducing neuronal apoptosis (Li et al., 2010; Jiang et al., 2014, 2015). Although XYW has been confirmed to affect multiple pathways that are targeted by antidepressants, the effect on oxidative stress remains unclear.

The NFE2L2/Kelch-like ECH associated protein-1 (KEAP1) pathway is a major regulator of redox homeostasis (Baird and Dinkova, 2011). NFE2L2 is usually retained in the cytosol, where it is tethered to its cytosolic repressor, KEAP1. A recent study has shown that NFE2L2 antioxidant signaling pathways are inhibited in the prefrontal cortex of patients with severe depression (Martín-Hernández et al., 2018). Furthermore, NFE2L2 gene knockout increases susceptibility to depression (Bouvier et al., 2017). NFE2L2 is also thought to be involved in the mechanisms underlying the antidepressant effect of serotonin reuptake inhibitors (Mendez-David et al., 2015). Taken together, it is evident that NFE2L2 plays an important role in the pathogenesis of depression (Martín-Hernández et al., 2016; Yao et al., 2016). Based on these findings, we hypothesize that long-term olfactory absence results in chronic stress and suppression of the NFE2L2 signaling pathway, which leads to the development of depression. However, the association between oxidative stress and the pathogenesis of depression is poorly understood, and there are currently no recognized therapies that effectively halt or slow the progression of depression. Therefore, using the OB rat model, we investigated whether XYW attenuated depression-like behaviors and oxidative stress. We also explored the mechanisms underlying these effects.

## Materials and Methods

### Xiaoyao Pills Quality Control

Xiaoyao Pills is composed of eight Chinese herbal medicines with the characteristics of complex composition. However, the Chinese Pharmacopoeia only provides content determination for paeoniflorin (C_23_H_28_O_11_). According to previous literature (Liu et al., 2018; Zhao et al., 2018), they analyzed the composition of XYW, including paeoniflorin, liquiritin, saikosaponin B2 and atractylenolide Ⅱ. In the present study, we determined the components of paeoniflorin (C_23_H_28_O_11_), liquiritin (C_21_H_22_O_9_), saikosaponin B2 (C_42_H_68_O_13_) and atractylenolide Ⅱ (C_15_H_20_O_2_).

The analysis was performed by high-performance liquid chromatography (HPLC) (Thermo, US). Hypersil GOLD^TM^C_18_ chromatographic column (250 mm × 4.6 mm, 5 μm, Thermo SCIENTIFIC) was used and the chromatographic separation conditions were as follows: mobile phase: 0.05% (V/V) phosphoric acid (A) + acetonitrile (B) (0∼30 min, 10∼25% B; 30–40 min, 25∼44% B; 40∼60 min, 44∼50% B; 60∼70 min, 50∼60% B; 70∼80 min, 60∼75% B; 80∼90 min, 75∼10% B; 90∼100 min, 10% B); detection wavelength: 230 nm (10∼16 min, paeoniflorin), 210 nm (16∼20 min, liquiritin), 210 nm (43∼47 min, saikosaponin B2), 230 nm (58∼62 min, atractylenolide II); column temperature: 30°C; flow rate: 1.0 ml·min^−1^; injection volume: 10 μL. Stock solutions of XYW was prepared by dissolving 1.0 g of analyte in 100 ml dilute methanol. The content of paeoniflorin (C_23_H_28_O_11_), liquiritin (C_21_H_22_O_9_), saikosaponin B2 (C_42_H_68_O_13_) and atractylenolide II (C_15_H_20_O_2_) in XYW was determined.

### Drugs and Reagents

The XYW (Tai Ji, China, batch number 1707029) and fluoxetine hydrochloride (FLX) (Patheon, France, 7686 A) were dissolved in pure water solution preparation (SOD, batch number 20180,309), malondialdehyde (MDA, batch number 20180,313) (GSH, batch number 20180,621), and GSH/glutathione disulfide (GSH/GSSG, batch number 20180,726) levels were assessed using commercially available kits (Nanjing Jiancheng Bioengineering Institute, Nanjing, China). Nitric oxide (NO) assay kit (Biyuntian, 0,72117,171,110, China). Rat interleukin 6 (IL6) ELISA Kit (MULTI SCIENCES, A30680231), rat interleukin 1β (IL1B) ELISA Kit (ExCell Bio, 21H183), rabbit anti-NTRK2 antibody (Cell Signaling Technology; Cat. No. #4603), rabbit anti-AKT1 antibody (Cell Signaling Technology; Cat. No. #4691S), rabbit anti-p-AKT1 antibody (Cell Signaling Technology; Cat. No. #4060S), rabbit anti-HMOX1 antibody (Cell Signaling Technology; Cat. No. #S2206S), rabbit anti-PIK3CA antibody (Servicebio; Cat. No. #LS190932), rabbit anti-GAPDH antibody (Servicebio; Cat. No. #GB11002), rabbit anti-SOD1 antibody (Novus Biologicals; Cat. No. #03253462C2B), rabbit anti-NFE2L2 antibody (Invitrogen; Cat. No. #TK2668681A), rabbit anti-brain-derived neurotrophic factor (BDNF) antibody (Abcam; Cat. No. #ab108319) and anti-rabbit IgG HRP-linked antibody (Servicebio; Cat. No. #GB23303).

### Animals

Male Sprague-Dawley rats weighing 180–200 g were purchased from Chengdu Dashuo Biotechnology Company (Chengdu, China, Qualified number: SCXK-[Chuan]-2015-030). Before commencing the experiment, the animals were randomly housed in cages at room temperature (25 ± 2°C) with a 12-h light/dark cycle. They had full access to food and water and were acclimatized to the environment for 3 days. The experiments were conducted in accordance with the guidelines of the Committee for Animal Care and Use of Laboratory Animals, College of Pharmacy, Chengdu University of Traditional Chinese Medicine (Chengdu, China).

### Olfactory Bulbectomy Surgery

**FIGURE 1:**
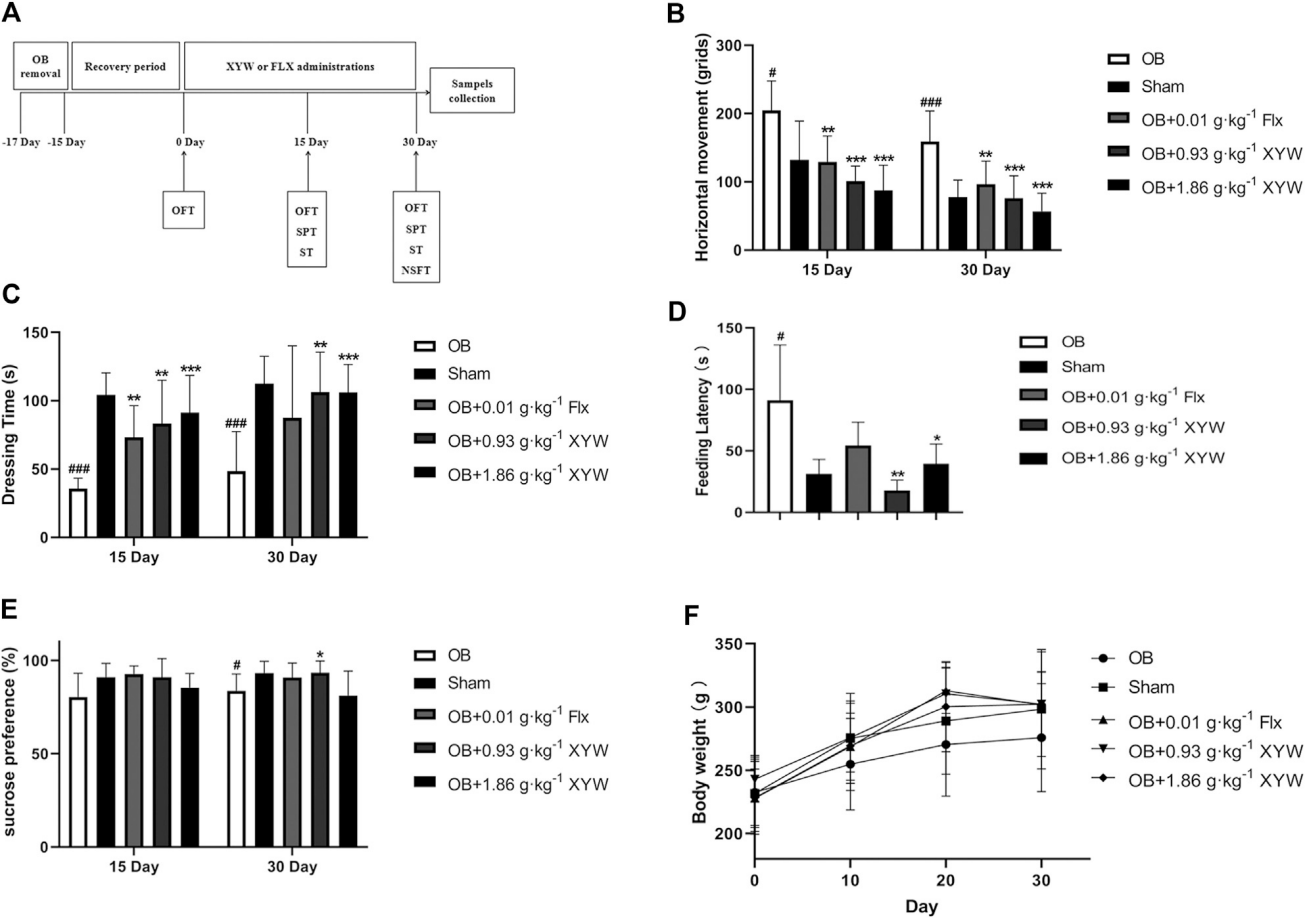
XYW administration reversed depression-like behaviors in OB rats **(A)** The program of OB model preparation and drug administration in the present study **(B)** Horizontal movement of rats in sham and OB groups in the presence or absence of FLX and XYW treatments in open filed test. *n* = 8 in each group **(C)** Dressing time in splash test. *n* = 8 **(D)** Feeding latency in novelty suppressed feeding test. *n* = 8 **(E)** Percentage of sucrose preference in sucrose preference test. *n* = 8 **(F)** Body weight. *n* = 8. Data are expressed as mean ± SD. #*p* < 0.05 *vs*. Sham group, *p < 0 0.05 and **p < 0.01 *vs*. OB group.

Olfactory bulbectomy was performed according to previously used methods (Van, 1990; Freitas et al., 2013) but with minor modifications. Briefly, rats were anesthetized, and the skull covering the olfactory bulbs was exposed using a midline incision. Then, 2 mm burr holes were drilled 8 mm anterior to the bregma and 2 mm lateral to the midline. The top of the rats’ heads was shaved and fixed to a brain stereotaxic instrument. The incisor bar was set at 4.5 mm below the interaural line. Both olfactory bulbs were obliterated and aspirated, the holes were filled with glass-ionomer cement, and the scalp was subsequently sutured. Sham-operated rats underwent the same procedure but without the bulb obliteration/aspiration. The animals received daily intramuscular injections of penicillin (8 × 105 U, 0.1 ml/200 g) for 3 days post-surgery to prevent infection and were subsequently housed individually in polypropylene cages. The experiments were continued after 15 days of recovery, after which the OB rats were selected using the open field test. The experimental procedure is illustrated in Figure 1A.

### Drug Treatment

Animals in the XYW group (0.93 and 1.86 g·kg^−1^) were intragastrically administered XYW daily for 30 days after the 15-days recovery from surgery. Animals in the FLX group (0.01 g·kg^−1^) were intragastrically administered FLX daily for 30 days after the 15-days recovery from surgery. Animals in the sham and OB groups received equivalent volumes of water daily to ensure isocaloric intake.

### Behavioral Studies

#### Open Field Test

The rats from each treatment group were put into the corner of an open box. After 2 min of adaptation, we observed and recorded the total distance traveled during a 4-min period (Zueger et al., 2005).

#### Sucrose Preference Test

Two water bottles were placed in each cage during feeding to eliminate the effect of animal habits on the experiment. Before testing, all rats were fasted and water-deprived for 24 h. On the day of testing, drinking water and a 2% sucrose solution were placed in the home cage for 6 h. At the end of testing, the fluid content was measured, and sucrose preference was calculated using the following equation: sucrose preference (%) = sucrose intake/(sucrose intake + water intake) × 100%

#### Splash Test

The splash test was performed in a standard rat cage with no bedding. The rats were sprayed with a 2 ml 10% sucrose solution on the dorsal while in their home cage to induce grooming behavior. We recorded the total grooming time during a 5-min period (Surget et al., 2008).

#### Novelty Suppressed Feeding Test

The rats were deprived of food for 48 h before testing. For the test, the rats were placed in an open field (60 × 60 × 20 cm) that had a small amount of food placed at the center. The time taken for the rat to bite the food was recorded as the feeding latency where rats were given up to 5 min (Wang et al., 2018b).

### Tissue Sample Collection

After the behavioral tests were completed, abdominal aorta bleeds were performed, and plasma was collected by centrifugation at 3,000 rpm for 15 min. Both the hippocampus and cortex tissues were immediately dissected on an ice-plate and frozen with liquid nitrogen. All tissue samples were then stored at −80°C.

### Oxidative Stress Assays

The levels of SOD, MDA, GSH, and GSH/GSSG in the serum and cortex were assessed using commercially available kits according to manufacturer instructions. Briefly, the cortex tissue was homogenized with 0.9% saline in an ice-bath and centrifuged (12,000 g, 4°C, 10 min). The supernatant and serum were applied to estimate the antioxidant content.

### Inflammatory Markers Assays

The levels of NO in the serum were determined using a commercially available NO assay kit according to manufacturer instructions. The optical density was measured at 540 nm using a micro-plate reader. The levels of IL6 and IL1B in the serum and cortex were determined using commercially available ELISA kits according to manufacturer instructions. Briefly, the cortex tissue was homogenized with 0.9% saline in an ice-bath and centrifuged (12,000 g, 4°C, 10 min). The supernatant and serum were applied to estimate the IL6 and IL1B content. Optical density was measured at 450 nm using a micro-plate reader.

### RNA Isolation and Gene Expression Assessment

Total RNA was used to synthesize complementary DNA using a FastQuant RT kit (Tiangen, Beijing, China). The amplification reactions were performed in 96-well reaction plates with a 20 μL reaction volume (Bio-Rad, Hercules, CA, United States). The gene primer sequences for Actb, Nfe2l2, Keap1, Gpx3, Hmox1, Nqo1, and Ogg1 used in this study are listed in Table 1. Data were normalized using the 2^−ΔΔCt^ method.

**TABLE 1:** Gene primer sequence.

Gene	Prime	Primer sequence (5′ to 3′)	Product size (bp)
Actb	Forward primer	CAC​CCG​CGA​GTA​CAA​CCT​TC	207
	Reverse primer	CCC​ATA​CCC​ACC​ATC​ACA​CC	
Nfe2l2	Forward primer	ATT​CCC​AGC​CAC​GTT​GAG​AG	128
	Reverse primer	TCC​TGC​CAA​ACT​TGC​TCC​AT	
Keap1	Forward primer	TCC​ATT​GAA​GGC​ATC​CAC​CC	117
	Reverse primer	GTA​CAT​GAC​TGC​CCC​GTT​CA	
Nqo1	Forward primer	CTC​AGG​TGG​CCT​GGG​ATA​TG	139
	Reverse primer	ACA​AGT​GGG​TGG​AGG​ATT​GG	
Gpx3	Forward primer	CGT​GAA​CGG​GGA​GAA​AGA​GC	198
	Reverse primer	CTG​ACT​GTG​GTC​CGG​TGG​TA	
Hmox1	Forward primer	CCA​TCC​CTT​ACA​CAC​CAG​CC	245
	Reverse primer	GCG​AGC​ACG​ATA​GAG​CTG​TT	
Ogg1	Forward primer	CCC​TCT​GGC​CAA​CAA​AGA​AC	167
	Reverse primer	GAT​CCC​TTT​TTG​CGC​TTT​GC	

### Western Blot Analysis

RIPA lysate buffer containing 1 mM phenylmethanesulfonyl fluoride and 1 mM phosphatase inhibitor cocktail was added to each sample to collect the total protein. The total protein concentration of each sample was determined using the bicinchoninic acid assay method, and all samples were adjusted to have the same concentration. The prtein samples were mixed with a 5× loading buffer and denatured at 95°C. The proteins were separated using sodium dodecylsulfonate-polyacrylamide gel electrophoresis and electrophoretically transferred onto polyvinylidene fluoride membranes. The membranes were probed with rabbit anti-NTRK2 (1:1000; Cell Signaling Technology; Cat. No. #4603), rabbit anti-AKT1 (1:1000; Cell Signaling Technology; Cat. No. #4691S), rabbit anti-p-AKT1 (1:1000-*Ak*; Cell Signaling Technology; Cat. No. #4060S), rabbit anti-HMOX1 (1:1000; Cell Signaling Technology; Cat. No. #S2206S), rabbit anti- PIK3CA (1:1000; Servicebio; Cat. No. #LS190932), rabbit anti-GAPDH (1:1000; Servicebio; Cat. No. #GB11002), rabbit anti-SOD1 (1:1000; Novus Biologicals; Cat. No. #03253462C2B), rabbit anti-NFE2L2 (1:1000; Invitrogen; Cat. No. #TK2668681A), and rabbit anti-BDNF (1:1000; Abcam; Cat. No. #ab108319) antibodies overnight at 4°C and then incubated with anti-rabbit IgG HRP-linked antibody (1:3000; Servicebio; Cat. No. #GB23303) at 37°C for 1.5 h. Detection was performed using a ChemiDoc XRS+ (BioRad, United States) image analysis system.

### Immunofluorescence Staining

The brain tissues were sliced into frozen sections (Thermo, Cryotome E), and the tissue sections were subsequently rewarmed. The tissue slides were incubated for 30 min at 37°C in the dark with DHE (Sigma, D7008) stain, which was diluted with phosphate-buffered saline. The slides were placed on a shaker and washed three times for 5 min each time. After drying the slide slightly, the DAPI dye solution was added dropwise, and the core was stained for 10 min at room temperature. The sections were observed under a fluorescence microscope (ECLIPSE TI-SR, Nikon, Japan), and the images were captured. The nucleus was stained blue, and the level of ROS in the cells was judged based on the intensity of red fluorescence. Image acquisition was performed at ×200 magnification. The relative expression of ROS was calculated according to the relative cumulative optical density (IOD), which was calculated by the formula: IOD = ROS cumulative optical density/nuclear stain cumulative optical density.

### Statistical Analyses

All statistical analyses were performed using SPSS 21 (IBM Corporation, Armonk, NY, United States). Data are presented as means ± standard deviations. All analyses were performed using a one-way analysis of variance (SNK was used for pairwise between group comparisons) or *t*-test. Differences were considered statistically significant at *p* < 0.05.

## Results

### Content of Paeoniflorin, Liquiritin, Saikosaponin B2 and Atractylenolide II in Xiaoyao Pills

**FIGURE 2:**
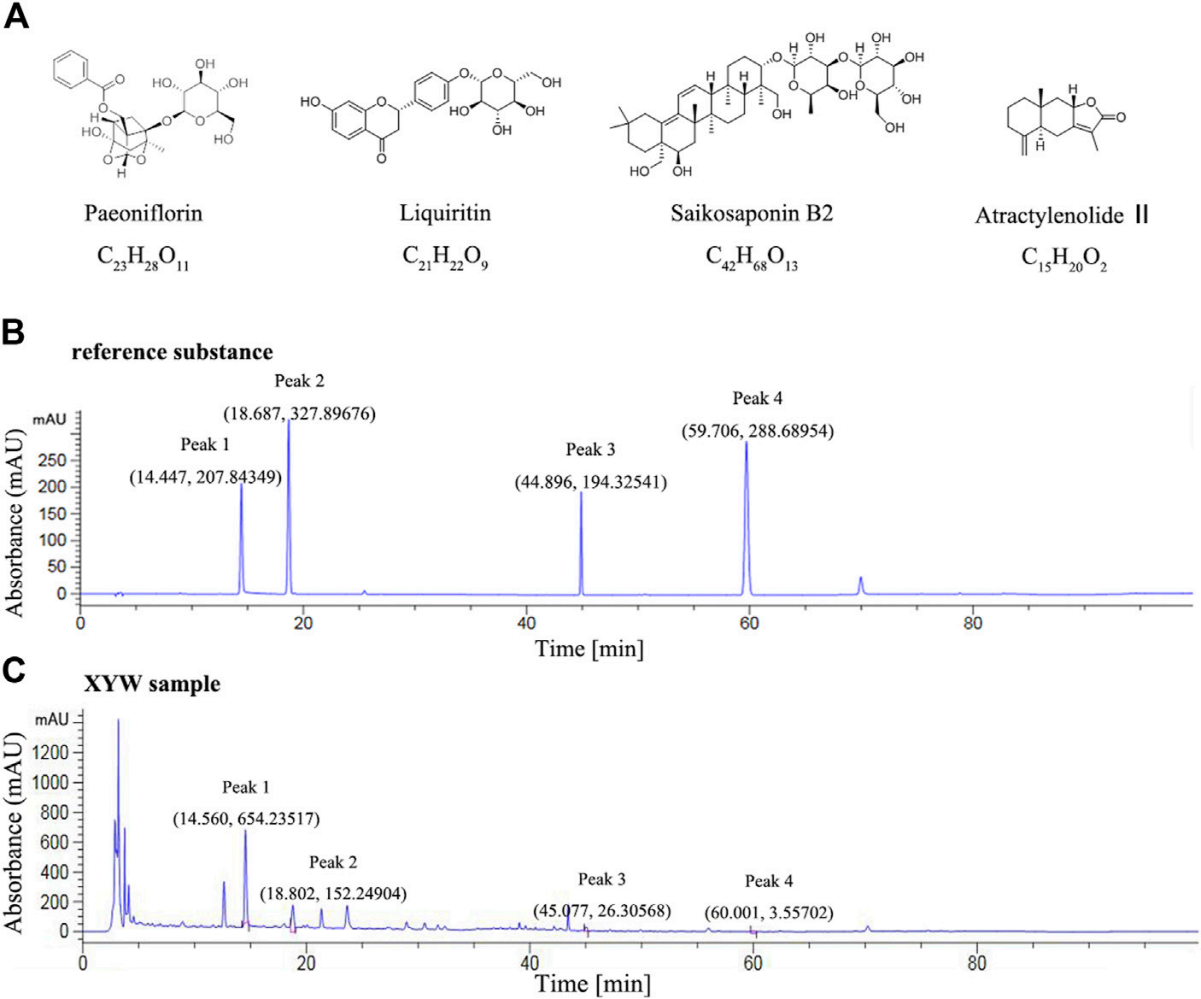
The quality control map of Xiaoyao Pills **(A)** The chemical structure formula of paeoniflorin, liquiritin, saikosaponin B2 and atractylenolide II **(B)** High performance liquid chromatography map of reference substance (Peak 1, 2, 3, 4 represent peak characteristic of paeoniflorin, liquiritin, saikosaponin B2, atractylenolide II, respectively) **(C)** High performance liquid chromatography map of Xiaoyao Pills sample (Peak 1, 2, 3, 4 represent peak characteristic of paeoniflorin, liquiritin, saikosaponin B2, atractylenolide II, respectively).

According to National Pharmacopoeia Committee (2020 edition), the content of paeoniflorin should not be less than 4.0 mg in 1.0 g of condensed pills. The characteristic map (chromatogram) of Xiaoyao Pills is shown in Figure 2, peak 1 represents peak characteristic of paeoniflorin, and the content of paeoniflorin in 1.0 g Xiaoyao Pills was 8.48 mg. Moreover, peak 2, 3, 4 represent peak characteristic of liquiritin, saikosaponin B2, atractylenolide II, respectively. The content of liquiritin, saikosaponin B2, and atractylenolide II in 1.0 g XYW is 1.31, 0.63, and 0.05 mg, respectively.

### Xiaoyao Pills Partially Reversed Depression-like Behaviors of Olfactory Bulbectomized Rats

On day 15, OB rats showed a significant increase in horizontal movements in the OFT (Figure 1B, *p* < 0.05) and a significant decrease in grooming time in the ST (Figure 1C, *p* < 0.001) compared with the sham group, whereas the FLX and XYW (0.93 and 1.86 g·kg^−1^) treatment groups showed a dramatic reversal of depression-like behaviors (*p* < 0.01 and *p* < 0.001, respectively).

In contrast with the sham group, on day 30, OB rats showed a significant increase in both horizontal movements in the OFT (Figure 1B, *p* < 0.001) and feeding latency in the NSFT (Figure 1D, *p* < 0.05), whereas grooming time in the ST (Figure 1C, *p* < 0.001) and sucrose preference in the SPT (Figure 1E, *p* < 0.05) decreased, which suggested that olfactory bulbectomy induced continuous depression-like behaviors. However, FLX treatment significantly alleviated the depression-like behavior of OB rats in the OFT only (Figure 1B, *p* < 0.05), whereas XYW (0.93 and 1.86 g·kg^−1^) treatment significantly reversed the depression-like behaviors in the OFT, ST, NSFT, and SPT (Figures 1B–E, *p* < 0.05, *p* < 0.01, and *p* < 0.001). Moreover, the trend of weight gain in OB rats was less than that in the sham rats, whereas the drugs restored that (Figure 1F). The behavioral results indicate that XYW (0.93 and 1.86 g·kg^−1^) can significantly improve the depression-like behaviors of rat caused by olfactory bulbectomy, suggesting that it has anti-depression-like effects.

### Xiaoyao Pills Mitigated Oxidative Stress and Neuroinflammation Caused by Olfactory Bulbectomy

**FIGURE 3:**
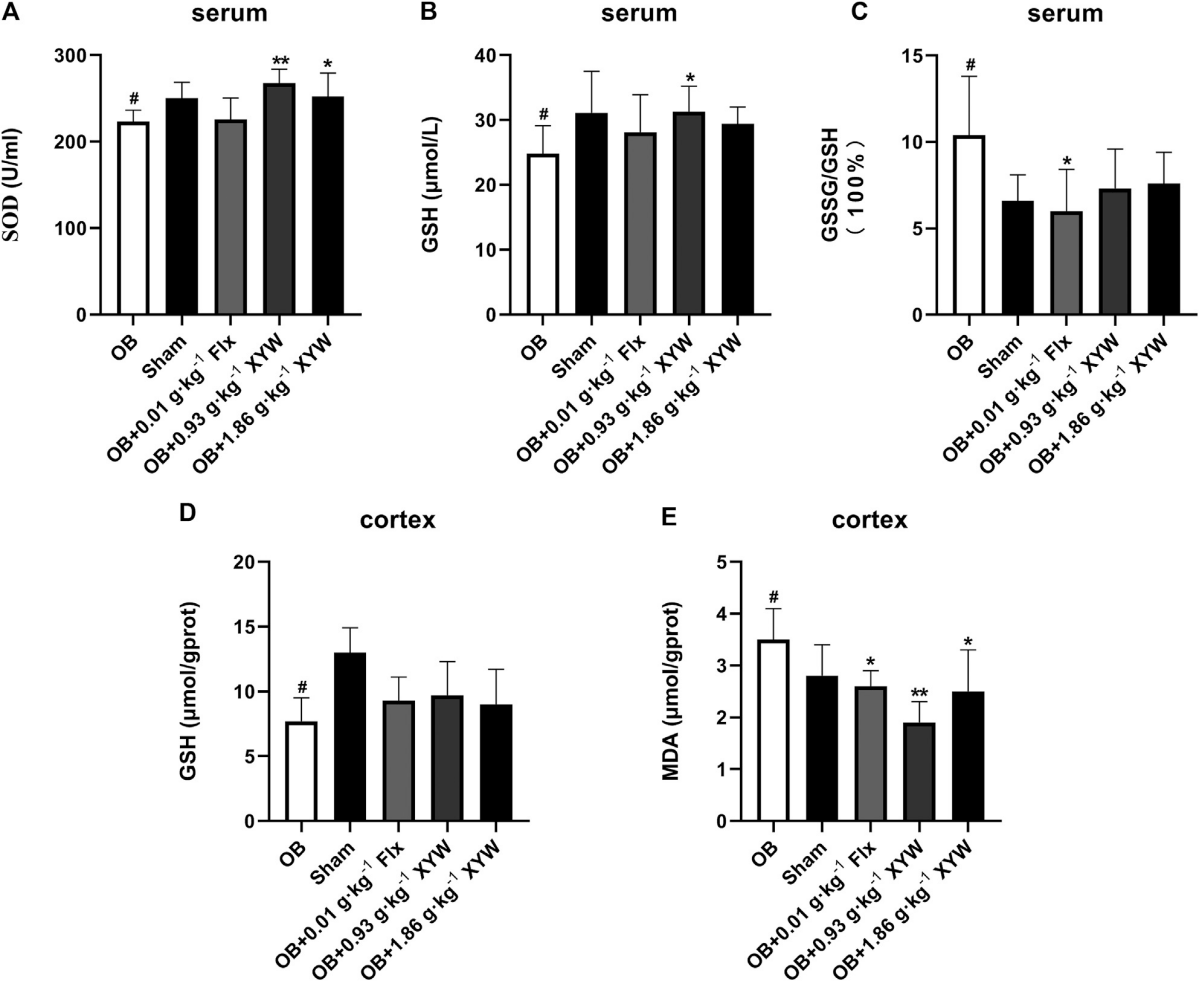
The anti-oxidative activities of XYW in OB model **(A and B, C)** In the serum, the activity of SOD and the levels of GSH and GSSG/GSH were reduced in OB rats, while XYW administration exhibited significantly more active of SOD and higher levels of GSH. *n* = 8 in each group **(D and E)** In the cortex, OB significantly affected the levels of GSH and MDA, while XYW administration significantly decreased the level of MDA. *n* = 8. Data are expressed as mean ± SD. #*p* < 0.05 *vs*. Sham group, *p < 0 0.05 and **p < 0.01 *vs*. OB group.

**FIGURE 4:**
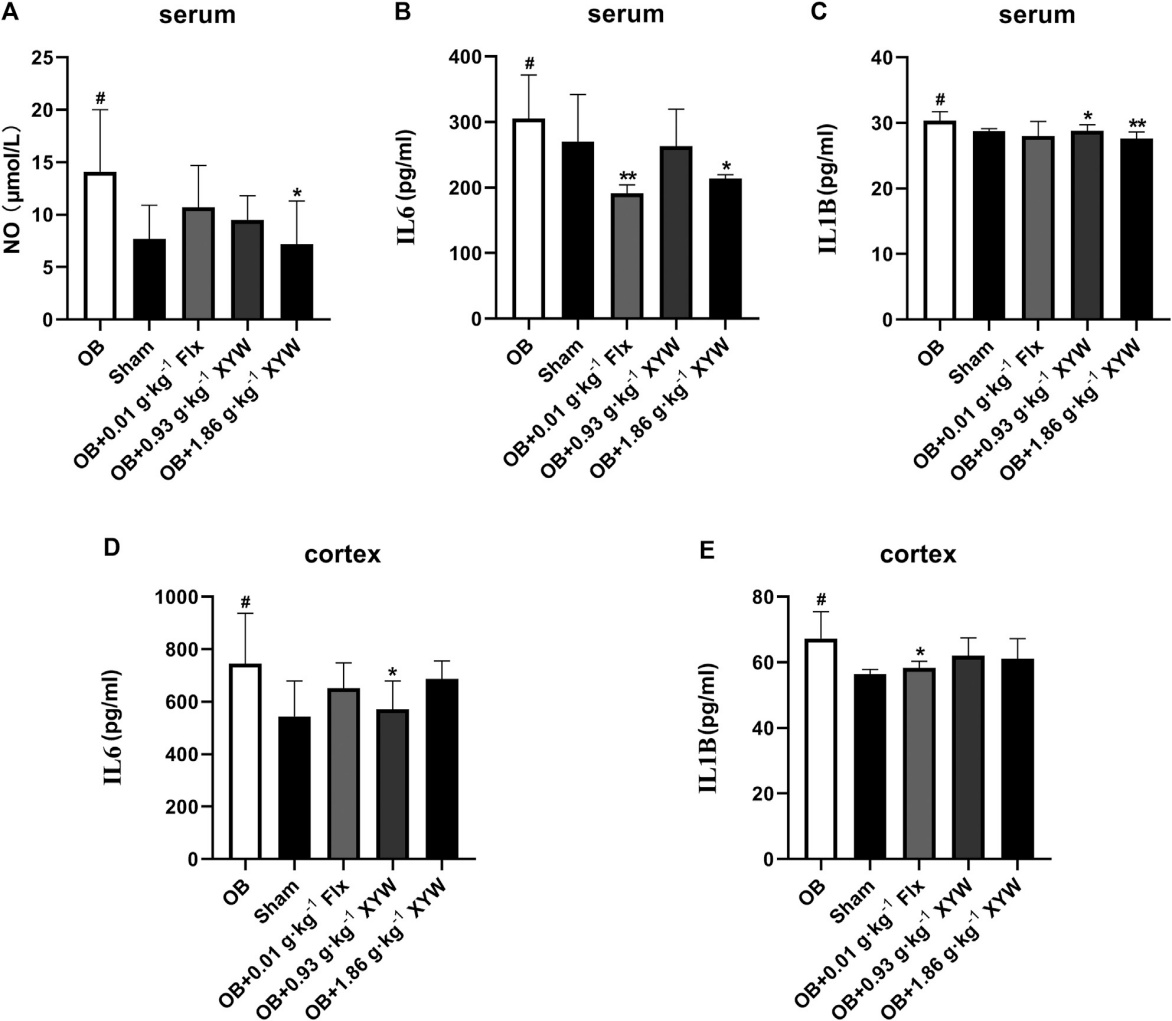
The anti-inflammatory activities of XYW in OB model **(A and** **B, C** **)** In the serum, the levels of NO, IL6 and IL1B were elevated in OB rats, while XYW administration exhibited significantly lower levels of NO, IL6 and IL1B. *n* = 5 in each group **(D and E)** In the cortex, OB significantly increased the levels of IL6 and IL1B, while XYW administration down-regulated the level of IL6. *n* = 5. Data are expressed as mean ± SD. ^#^ *p* < 0.05 *vs*. Sham group, *p < 0 0.05 and **p < 0.01 *vs*. OB group.

To investigate whether olfactory bulbectomy caused oxidative stress, we assessed the relevant antioxidants. As shown in Figures 3A–C, both the SOD activity and GSH level in the serum of OB rats were significantly lower than those of the sham rats (*p* < 0.05), whereas the ratio of GSSG/GSH was significantly higher (*p* < 0.05); these were significantly alleviated by treatment with FLX and XYW (0.93 and 1.86 g·kg^−1^). Similarly, the OB rats exhibited lower GSH activity (*p* < 0.05) and higher MDA (*p* < 0.05) levels in the cortex (Figures 3D,E); treatment with FLX (*p* < 0.05) and XYW (1.86 g·kg^−1^, *p* < 0.05; 0.93 g·kg^−1^, *p* < 0.01) effectively reduced MDA levels without reversing the levels of GSH. Our examination of the production of cytokines to detect whether further inflammation occurred due to oxidative stress revealed significant elevation in the levels of NO, IL6, and IL1B in both the serum and cortex of OB rats. Moreover, treated rats also showed alleviation and enhanced expression of the inflammatory mediator, NO, and proinflammatory cytokines, IL6 and IL1B (Figures 4A–E).

Taken together, these results confirm that XYW (0.93 and 1.86 g·kg^−1^) administration can restore the damage caused by central and peripheral oxidative stress and minimize further inflammation, which demonstrates the neuroprotective effect of XYW.

### Xiaoyao Pills Reduced the Production of ROS in the Cortex and Hippocampus of Olfactory Bulbectomized Rats

**FIGURE 5:**
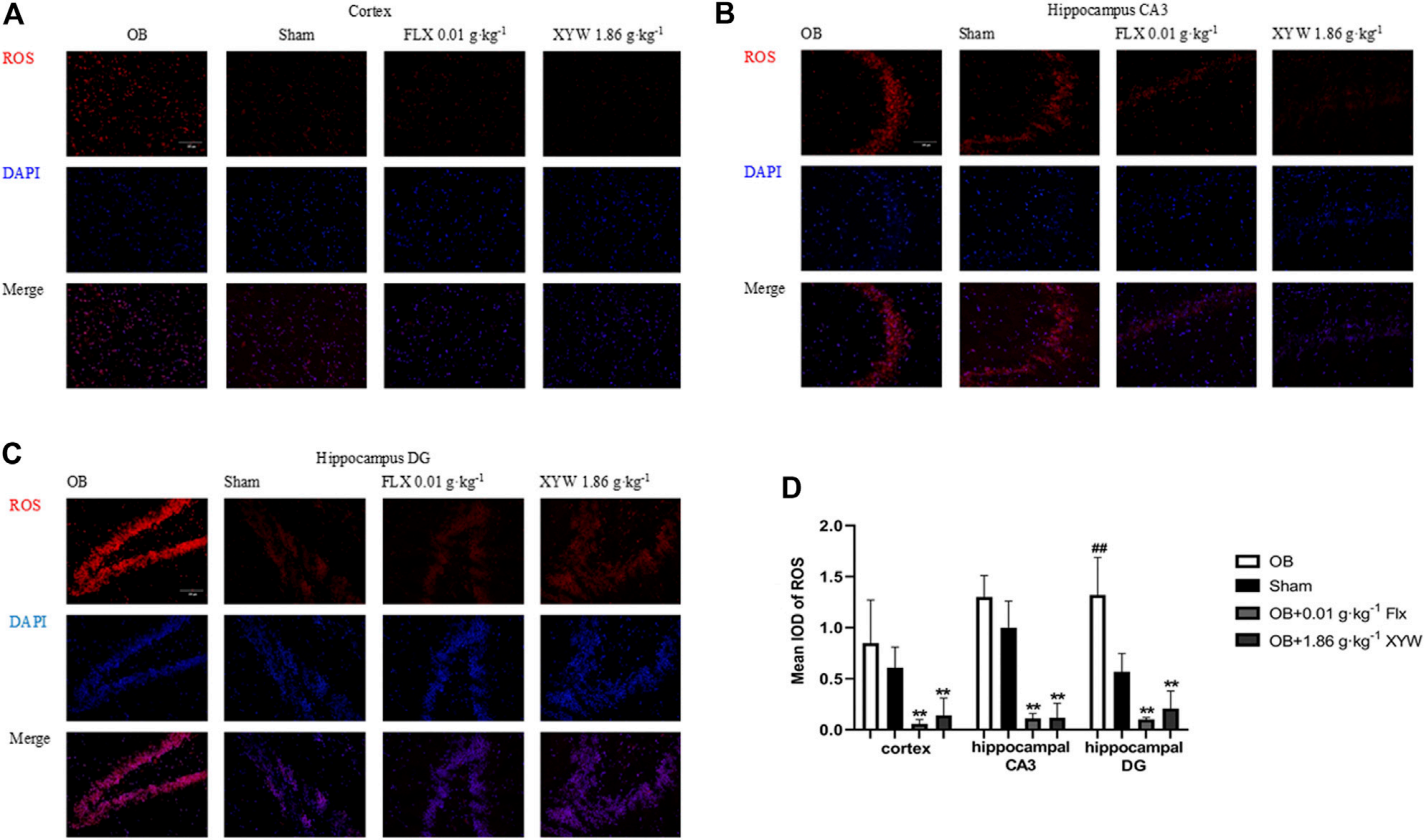
XYW reduced the production of ROS in the cortex and hippocampus CA3/DG regions of OB rats **(A and** **B, C** **)** Immunofluorescence of ROS in rat brain (×200) **(A)** Cortex. *n* = 3 in each group **(B)** *Hippocampus* CA3 region. *n* = 3 **(C)** *Hippocampus* DG region. *n* = 3 **(D)** Mean iod of ROS. Data are expressed as means ± SD. ##*p* < 0.01 *vs*. Sham group, **p < 0 0.01 *vs*. OB group.

An increased ROS level is an important indicator of oxidative stress. To visually observe oxidative stress levels, we conducted immunofluorescence detection of ROS in the cortex and CA3 and DG regions of the hippocampus. A DHE dye solution was used to tag superoxide anion (${\text{O}}_{2}^{-}$), which is shown by the red ROS staining in Figures 5A–C. As shown in Figure 5D, OB rats showed slightly more ROS fluorescence in the cortex and hippocampal CA3 region than the sham rats and had significantly stronger ROS fluorescence signals in the hippocampal DG region (*p* < 0.01). Similarly, XYW (1.86 g·kg^−1^) and FLX treated rats showed distinctly less red fluorescence in the cortex and hippocampal CA3 and DG regions (*p* < 0.01). Our results further indicate that high doses of XYW eliminate ROS to reduce the oxidative stress level in the cortex and hippocampus of OB rats.

### Xiaoyao Pills Restored the Gene Transcription and Protein Expression of Nfe2l2/Keap1 Pathway in Olfactory Bulbectomized Rats

It is well known that the Nfe2l2/Keap1 pathway, which promotes transcription of antioxidants and reduces oxidative stress damage, is of vital importance for the antioxidative system. Therefore, we further studied gene transcription and protein expression of the Nfe2l2/Keap1 pathway in the OB rats.

**FIGURE 6:**
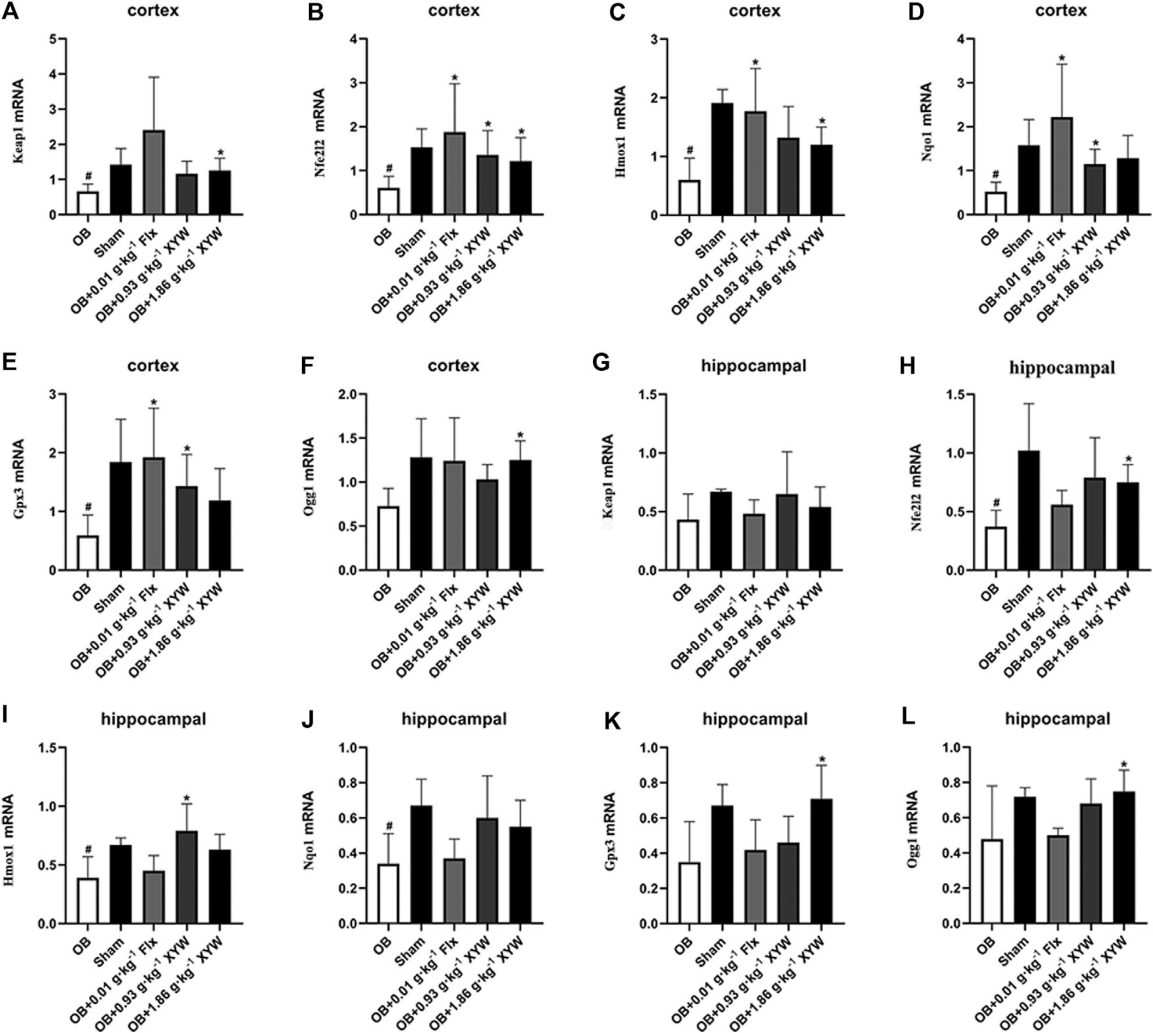
XYW up-regulated the Nfe2l2/Keap1 pathway and related antioxidant enzymes genes expression in the cortex and hippocampus of OB rats **(A–F)** Genes expression of Keap1, Nfe2l2, Hmox1, Nqo1, Gpx3 and Ogg1in the cortex. n = 4 in each group **(G–L)** Genes expression of Keap1, Nfe2l2, Hmox1, Nqo1, Gpx3 and Ogg1 in the hippocampus. n = 4. Data are expressed as means ± SD. #*p* < 0.05 *vs*. Sham group, *p < 0 0.05 *vs*. OB group.

As shown in Figures 6A–E, the messenger RNA (mRNA) levels of Nfe2l2 and Keap1 as well as the downstream Hmox1, Nqo1 and Gpx3 (*p* < 0.05) were significantly downregulated (*p* < 0.05) in the cortex of OB rats. Consistent with previous findings, treatment with FLX and XYW (0.93 and 1.86·g kg^−1^) showed marked antioxidant effects. FLX treatment significantly restored transcription of Nfe2l2, Hmox1, Nqo1 and Gpx3 mRNA (*p* < 0.05), and XYW (0.93 and 1.86 g·kg^−1^) treatment restored transcription of Nfe2l2, Keap1, Hmox-1, Nqo1 and CPX3 mRNA (*p* < 0.05). In the hippocampus of OB rats (Figures 6G–K), there was a significant reduction of Nfe2l2, Hmox1 and Nqo1 mRNA (*p* < 0.05), whereas the downregulated Hmox1 and Nfe2l2 mRNA were restored by XYW (0.93 and 1.86 g·kg^−1^) treatment (*p* < 0.05). Furthermore, Ogg1 (Figures 6F,L), a specific enzyme that repairs DNA oxidative damage, was decreased in both the cortex and hippocampus of OB rats and was significantly improved by XYW (1.86 g·kg^−1^) treatment (*p* < 0.05).

**FIGURE 7:**
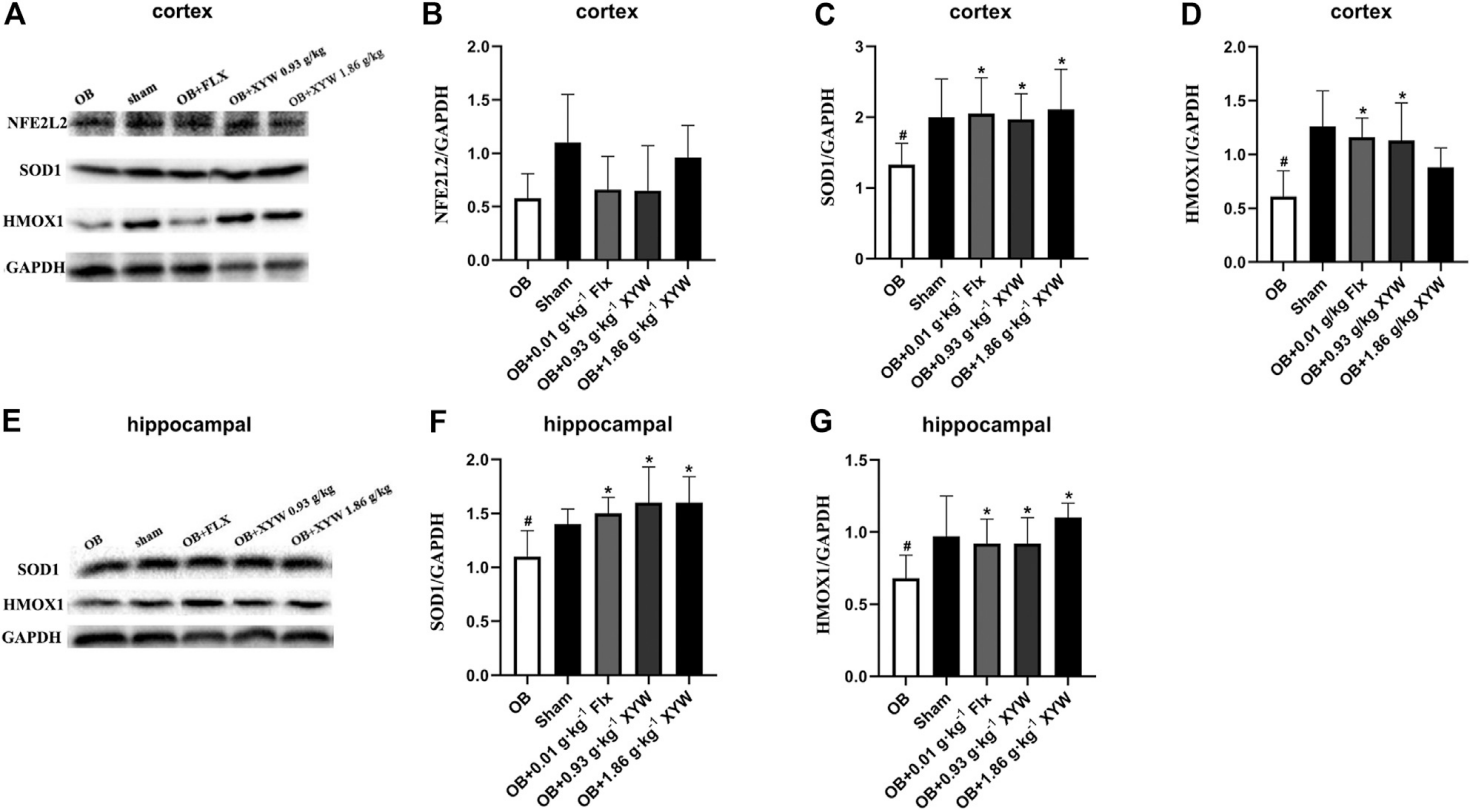
XYW activated NFE2L2/KEAP1 pathway both in cortex and hippocampus of OB rats **(A and** **B, C, D** **)** In the cortex, the protein expression levels of SOD1 and HMOX1 were significantly down-regulated in OB rats, while XYW administration markedly exhibited higher levels of SOD1 and HMOX1. *n* = 5 in each group **(E and** **F, G** **)** In the hippocampus, XYW observably reversed olfactory bulbectomy evoked decreasing levels of SOD1 and HMOX1. *n* = 5. Data are expressed as mean ± SD. #*p* < 0.05 *vs*. Sham group, *p < 0 0.05 *vs*. OB group.

In terms of protein level, HMOX1 and SOD1 were significantly reduced in the cortex and hippocampus of OB rats (*p* < 0.05), whereas FLX and XYW (0.93 and 1.86 g·kg^−1^) treatments accelerated the production of HMOX1 and SOD1 (*p* < 0.05; Figures 7A–G). As shown in Figures 7A,B, the downregulation of NFE2L2 in the cortex was reversed by XYW (1.86 g·kg^−1^) treatment; however, this was not statistically significant. These findings suggest that during states of excessive oxidative stress, the NFE2L2/KEAP1 pathway is suppressed, so that downstream antioxidants, such as HMOX1, NQO1, GPX3 and SOD1 are reduced, which heightens the risk for DNA damage. Moreover, XYW treatments reverse the downregulated NFE2L2/KEAP1 pathway to produce central antioxidant effects.

### Xiaoyao Pills Restored the Suppressed Upstream Protein Expression of the PIK3CA/AKT1 Pathway in Olfactory Bulbectomized Rats

**FIGURE 8:**
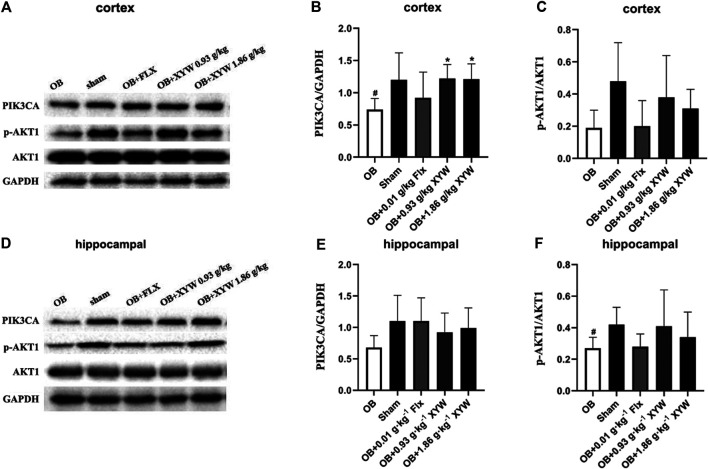
XYW activated PIK3CA/AKT1 pathway both in cortex and hippocampus of OB rats **(A and** **B, C** **)** In the cortex, the levels of PI3K significantly decreased and the ratio of *p*-AKT1/AKT1 tended to reduce in the OB rats while XYW administration significantly restored the reduction of PIK3CA and *p*-AKT1/AKT1 in OB rats. n = 5 in each group **(D and** **E, F** **)** Similarly, in the hippocampus, XYW tended to reverse olfactory bulbectomy caused decreasing in the level or ratio of PIK3CA and *p*-AKT1/AKT1. *n* = 5. Data are expressed as mean ± SD. #*p* < 0.05 *vs*. Sham group, *p < 0 0.05 *vs*. OB group.

With mild levels of oxidative stress, the production of ROS activates the PIK3CA/AKT1 pathway and promotes the cleavage of NFE2L2/KEAP1 and nuclear translocation of NFE2L2, which activates the production of downstream antioxidant enzymes, reducing ROS levels to achieve an antioxidant effect (Cameron et al., 2018; Li et al., 2018). However, many investigations have found that ROS inhibit the activation of the PIK3CA/AKT1 pathway, which exacerbates oxidative stress and inflammation, leading to excessive oxidative damage (Wang K. S. et al., 2017; Jiang et al., 2018). In the present study, olfactory bulbectomy led to the suppression of the NFE2L2/KEAP1 pathway, so we assessed protein expression of the PIK3CA/AKT1 pathway to study whether changes in the PIK3CA/AKT1 pathway were similar to those in the downstream NFE2L2/KEAP1 pathway. As shown in Figures 8A–C, the expression of PIK3CA in the cortex of OB rats was significantly lower than that of the sham rats (*p* < 0.05), whereas the expression of AKT1 showed a decreasing trend. Similarly, PIK3CA in the hippocampus of OB rats showed a decreasing trend and AKT1 was significantly lower (*p* < 0.05) compared with the sham rats (Figures 8D–F). We also found that administering FLX and XYW (0.93 and 1.86 g·kg^−1^) improved the protein levels of PIK3CA and AKT1 to some extent (Figures 8D–F). Our results show that the PIK3CA/AKT1 pathway that is upstream of the ROS and NFE2L2/KEAP1 pathways is suppressed as well in the central because of the damage due to the oxidative stress caused by olfactory bulbectomy; however, this could be restored by XYW treatment.

### Xiaoyao Pills Restored the Decreased Protein Expression of the NTRK2/BDNF Pathway in Olfactory Bulbectomized Rats

**FIGURE 9:**
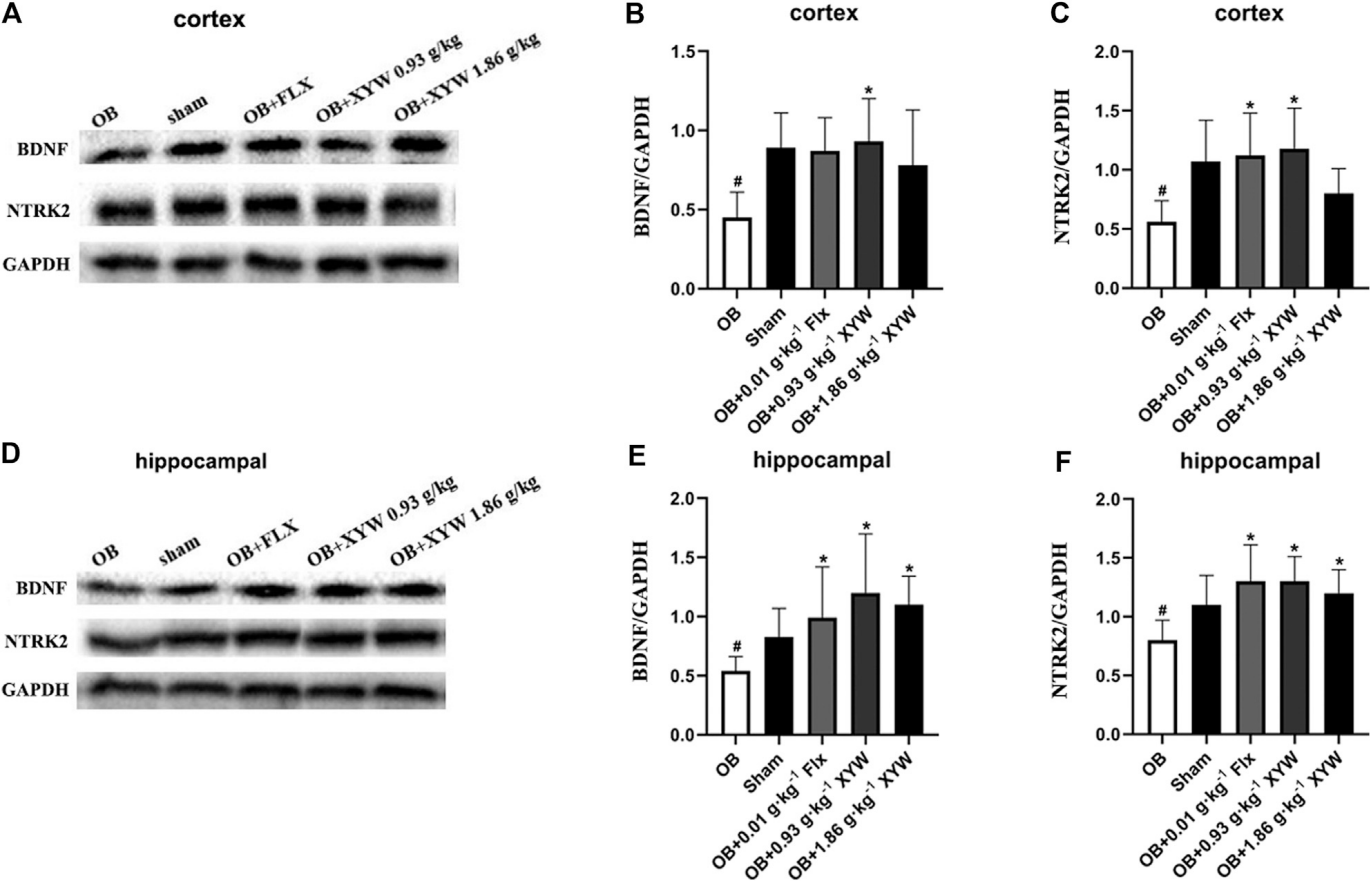
XYW activated NTRK2/BDNF pathway both in cortex and hippocampus of OB rats **(A and** **B, C** **)** In the cortex, the levels of BDNF and NTRK2 were significantly down-regulated in OB rats, while XYW administration markedly exhibited higher levels of BDNF and NTRK2. *n* = 5 in each group **(D and** **E, F** **)** In the hippocampus, XYW observably reversed olfactory bulbectomy evoked decreasing levels of BDNF and NTRK2. *n* = 5. Data are expressed as mean ± SD. #*p* < 0.05 *vs*. Sham group, *p < 0 0.05 *vs*. OB group.

BDNF has been well established as a specific indicator of depression-related diseases. Furthermore, the production of BDNF is closely related to the activation of the PIK3CA/AKT1 and CREB1/BDNF pathways (Mohammadi et al., 2018; Rai et al., 2019). The activation of PIK3CA induces the phosphorylation of AKT1, which activates CREB1, resulting in the elevation in the expression of BDNF. Moreover, the binding of NTRK2 and BDNF activates the PIK3CA/AKT1 pathway, which promotes synaptic plasticity and enhances long-term potentiation to alleviate depression. Because the PIK3CA/AKT1 pathway was suppressed due to excessive oxidative stress, we tested whether the expression of BDNF and the activity of the downstream pathway were also inhibited. As shown in Figures 9A–F, the protein levels of NTRK2 and BDNF in both the cortex and hippocampus of the OB rats were significantly downregulated compared with that of the sham rats (*p* < 0.05). FLX and XYW (0.93 and 1.86 g·kg^−1^) treatment dramatically restored the reduction in protein expression of NTRK2 and BDNF (*p* < 0.05), which demonstrated the neuroprotective effects of the treatments. Our results suggest that oxidative stress damage caused by olfactory bulbectomy is accompanied by the suppression of the NTRK2/BDNF pathway, and XYW is able to reverse this effect to promote the expression of BDNF and provide neuroprotection.

## Discussion

**FIGURE 10:**
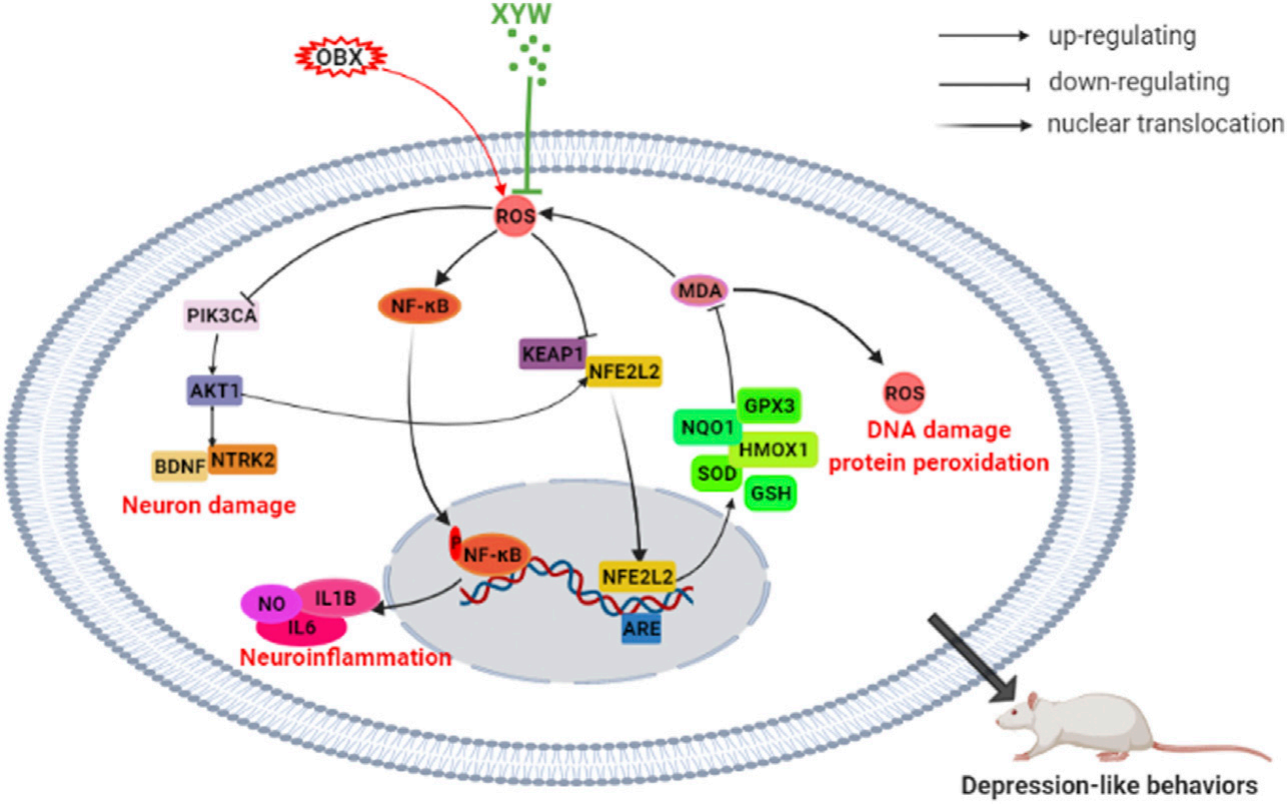
XYW alleviated olfactory bulbectomy induced depression-like behaviors via activating PIK3CA-AKT1-NFE2L2/BDNF pathway in the cortex and hippocampus.

Depression is a common chronic disease, which seriously affects the physical health and quality of life of patients. XYW, a clinically prescribed medication, has been shown to induce specific antidepressant effects; however, the underlying mechanism remains unclear. Olfactory bulbectomy is a recognized animal model of depression that induces changes in physiological functions and behaviors in the animal that resemble symptoms commonly observed in patients with depression (Czéh et al., 2016). Our findings reveal that XYW effectively ameliorate the behavioral dysfunctions induced by olfactory bulbectomy through the attenuation of oxidative stress and neuroinflammation by activating the PIK3CA-AKT1-NFE2L2/BDNF pathways in the cortex and hippocampus. A schematic representation of the underlying mechanism of the antidepressant effects of XYW are illustrated in Figure 10.

Previous reports have confirmed that neuroinflammation is involved in the pathogenesis of depression and may be a potential therapeutic target for depression (Dowlati et al., 2010; Marc et al., 2014). In our OB rats, high levels of ROS expression were found in the hippocampal DG and CA3 regions and cortex, and MDA levels were elevated in the cortex, which suggests that the OB rats experienced oxidative damage. In addition, the high GSSG/GSH ratio and reduced activity of SOD in the serum indicated that OB rats had an impaired antioxidant system, which weakened their ability to scavenge for oxygen-free radicals. Moreover, the imbalance of redox homeostasis correlated with depression. Evidence has confirmed that high levels of ROS can act as upstream messengers to activate the NLRP3 (Suárez and Buelvas, 2015) and NF-κB inflammatory signaling pathways (Formentini et al., 2017; Gan et al., 2017). Furthermore, the production of caspase-1 induces the maturation of the inflammatory mediator IL1B (Du et al., 2016). Consequently, oxidative stress in the central may cause neuroinflammation, which is an important factor that contributes to neuronal damage.

As mentioned earlier, NFE2L2 has been implicated in various psychiatric disorders, such as depression and anxiety (Hashimoto, 2018; Perić et al., 2017). Activation of the NFE2L2 signaling pathway can improve the lipopolysaccharide (LPS)-induced inflammatory response and reduce damage due to oxidative stress (Ren et al., 2019). In the LPS and chronic unpredictable mild stress-induced depression models, NFE2L2 signals were found to play key roles (Huang et al., 2013; Yang et al., 2018b). The NFE2L2-ARE pathway is an intrinsic mechanism of defense against oxidative stress. Moreover, NFE2L2 is a transcription factor that induces the expression of a large number of cytoprotective and detoxification genes (Buendia et al., 2016). During states of adequate oxidative stress, the NFE2L2 detaches from KEAP1 (an endogenous inhibitor of NFE2L2) and transfers to the nucleus to combine with ARE, which promotes the translation of antioxidants, such as SOD and HMOX1 (Tu et al., 2019). In the present study, the expression of NFE2L2 and KEAP1 as well as downstream antioxidant enzymes (e.g., GPX3, NQO1, and HMOX1) were significantly reduced in OB rats. We also observed a simultaneous increase in IL6 and IL1B levels in both the serum and cortex, which indicated that sustained olfactory bulbectomy-induced oxidative stress triggered an inflammatory response, which inhibited the activation of the NFE2L2 antioxidant signaling pathway. Notably, XYW treatment significantly lowered the levels of MDA, ROS, NO, IL6, and IL1B, elevated the levels of GSH, SOD, GPX, NQO1, HMOX1, NFE2L2, and KEAP1, and ameliorated the behavioral deficits that resulted from olfactory bulbectomy. Recent studies have confirmed that XYW mitigates LPS-induced neuroinflammation, synaptic damage, and neuron deficiency both *in vivo* and *vitro* (Shi et al., 2019a; Fang et al., 2020). These results suggest that XYW regulates oxidative stress and neuroinflammation via the NFE2L2/KEAP1 pathway.

The PIK3CA/AKT1 pathway is a core component in the pathogenesis of depression. Firstly, the PIK3CA/AKT1 pathway plays an essential role in the uptake and transport of serotonin, glutamate, and acetylcholine, and thus regulates the level and function of neurotransmitter receptors. Secondly, PIK3CA catalyzes PIP2 into PIP3, which induces the phosphorylation of AKT1. Subsequently p-AKT1 activates several downstream pathways, such as the mTOR, FoxO, GSK-3β, and NF-κB pathways, thereby regulating protein synthesis, cell proliferation, differentiation, autophagy, and apoptosis (Magariños et al., 1996; Polter et al., 2009; Li et al., 2010; Beurel et al., 2011; Dou et al., 2015). Thirdly, the PIK3CA/AKT1 pathway can cease ROS production by facilitating the synthesis of SOD and promoting appropriate mitochondrial autophagy, leading to an improvement mitochondrial function (Cunningham et al., 2007; Bellot et al., 2009). In our study, we found that olfactory bulbectomy caused mass production of ROS in the cortex and hippocampus, which inhibited the PIK3CA-AKT1-NFE2L2/KEAP1 pathway, leading to reduced production of antioxidant enzymes and weakened clearing capacity of ROS in the central. Finally, the PIK3CA/AKT1 pathway interacts with BDNF, as was demonstrated in our study. On the one hand, phosphorylation of AKT1 can activate CREB, which further promotes the synthesis of BDNF (Jiang et al., 2017). On the other hand, when BDNF binds with NTRK2, a BDNF specific receptor, the PIK3CA/AKT1 pathway is activated (Jiang et al., 2017), which retriggers the physiological process. We also found that there was reduced expression of BDNF and NTRK2 in the cortex and hippocampus of OB rats, which may have been due to oxidative damage, and indicated that the development and nutrition of neurons were compromised. Overall, our study verified that during states of excessive oxidative stress, the PIK3CA/AKT1/NFE2L2/KEAP1 and PIK3CA/AKT1/BDNF/NTRK2 signaling pathways are suppressed, which induced OB rats to exhibit phenotypic behaviors of depression.

In recent decades, there has been limited improvement in the drugs used to treat depression, with selective serotonin reuptake inhibitors (SSRIs) continuing to be the most widely used class of antidepressants (Berton and Nestler, 2006). However, SSRIs generally take several weeks to achieve a therapeutic effect. It has been reported than about a third of patients do not respond to SSRIs; moreover, SSRIs can have side effects in some patients (Rush et al., 2006; Holtzheimer and Mayberg, 2011). The disadvantages of SSRI treatment are mainly attributed to the unclear pathogenesis of depression. Therefore, further studies on the pathogenesis of depression is necessary to develop more effective treatments. Several studies to date have shown that the pathogenesis of depression is complex and involves multi-target and multi-pathway regulation. Complex components and difficulties in identifying effective components are the disadvantages of traditional Chinese medicine; although, they are also the advantages of the treatment of depression. For example, there are numerous representative effective constituents of XYW that have antidepressant effects: Total Saikosaponins, which has anti-inflammation, anti-apoptosis, and synaptic protein expression promoting effects (Lixing et al., 2018; Sun et al., 2018); Glycyrrhizic acid, which has an antidepressant effect by modulating autophagy, promoting anti-inflammation, and ameliorating the kynurenine pathway (Wang et al., 2018a; Yang et al., 2018a; Cao et al., 2020); and Paeoniflorin, which exerts a neuroprotective effect by inhibiting oxidative stress and Ca^2+^ overload, promotes neurogenesis in the hippocampal dentate gyrus, and activatess the MAPK1-CREB1 pathway (Mao et al., 2010; Chen et al., 2019; Zhong et al., 2019). These three components have varying mechanisms of action to induce the antidepressant effect, which suggests that the advantage of TCM compounds as depression treatments lies in the complexity of its components; therefore, TCMs have the potential to restore molecular imbalances at different levels in a multi-component and multi-target manner. As shown in our previous studies, Xiaoyao powder and XYW may offer an effective form of depression treatment by improving neurotransmission, neuroinflammation, neurogenesis, and other aspects in multi-component, multi-target and multi-pathway ways. Moreover, XYW can be extensively used to treat a variety of uncomfortable symptoms caused by depression, such as decreased gastrointestinal function, decreased sexual desire, and insomnia (Gong, 2014; Cao et al., 2019; Xian, 2007). These benefits are related to its effects of smoothing the liver, nourishing the blood, and strengthening the spleen, which are effects that cannot be achieved by FLX.

Both the cortex and hippocampus are two important brain regions that are associated with the pathogenesis of depression. The cortex is a significant nerve center that regulates thinking and behavior, whereas the hippocampus is responsible for memory formation, storage, and emotional adjustment (Treadway et al., 2015; Hainmueller and Bartos, 2018). Recent studies have shown that stress and depression have an impact on cortical and hippocampal morphology (Belleau et al., 2019) and function, such as suppressed neuroplasticity, decreased spine density, downregulation of neurotropic factors, and increased neuroinflammation (Lanz et al., 2019; Zhang et al., 2014; Giridharan et al., 2019). In the present study, we found that XYW promoted transcription of KEAP1/NFE2L2 pathway genes and upregulated protein expression of NFE2L2, SOD1, HMOX1, p-PIK3CA, p-AKT1, BDNF, and NTRK2 in both the cortex and hippocampus, which suggests that XYW has antioxidative and neuroprotective effects on both regions.

Overall, we demonstrated that by removing the olfactory bulb, it is possible to induce continuous oxidative stress in the cortex and hippocampus, which results in increased levels of ROS and MDA, reduced activity of SOD, HMOX1, NQO1, GSH, and GPX3, and increased lipid peroxidation. Moreover, we find that the PIK3CA/AKT1, NFE2L2/KEAP1, and NTRK2/BDNF pathways are suppressed, whereas XYW induces the clearance of excessive ROS and enhances the activation of the PIK3CA-AKT1/NFE2L2/BDNF pathway, which results in antidepressant and neuroprotective effects. Although determining the underlying mechanisms requires further investigation, our research highlights that NFE2L2 antioxidant signaling is important in depression and raises the possibility that the Chinese herbal compound, XYW, has promise as an effective treatment for depression.

## Conclusion

Since there may be a risk of non-specific effects at this dose level, it will be important to further study this extract at lower dose levels.

## Data Availability

The raw data supporting the conclusions of this article will be made available by the authors, without undue reservation, to any qualified researcher.

## Abbreviations

ACTB, actin beta; AKT1, Akt1 serine/threonine kinase 1; BDNF, brain derived neurotrophic factor; CREB1, cAMP responsive element binding protein 1; FLX, fluoxetine hydrochloride; GAPDH, glyceraldehyde-3-phosphate dehydrogenase; GPX3, glutathione peroxidase 3; GSH, glutathione; GSSH, glutathione (Oxidized); HMOX1, heme oxygenase 1; IL6, interleukin 6; IL1B, interleukin 1 beta; KEAP1, kelch like ECH associated protein 1; MDA, malondialdehyde; NO, nitric oxide; MAPK1, mitogen-activated protein kinase 1; NFE2L2, nuclear factor, erythroid 2 like 2; NQO1, NAD(P)H quinone dehydrogenase 1; NLRP3, NLR family pyrin domain containing 3; NSFT, novelty suppressed feeding test; OFT, open field test; OGG1, 8-oxoguanine DNA glycosylase; PIK3CA, phosphatidylinositol-4,5-bisphosphate 3-kinase catalytic subunit alpha; ROS, reactive oxygen species; ST, splash test; SPT, sucrose preference test; SOD, superoxide dismutase; NTRK2, neurotrophic receptor tyrosine kinase 2; XYW, xiaoyao pills.
